# NMR-Based Metabolomic Comparison of *Brassica oleracea* (Var. *italica*): Organic and Conventional Farming

**DOI:** 10.3390/foods9070945

**Published:** 2020-07-17

**Authors:** Massimo Lucarini, Maria Enrica Di Cocco, Valeria Raguso, Flavia Milanetti, Alessandra Durazzo, Ginevra Lombardi-Boccia, Antonello Santini, Maurizio Delfini, Fabio Sciubba

**Affiliations:** 1 CREA—Research Centre for Food and Nutrition, Via Ardeatina 546, 00178 Rome, Italy; alessandra.durazzo@crea.gov.it (A.D.); g.lombardiboccia@crea.gov.it (G.L.-B.); 2 Department of Chemistry, “Sapienza” University of Rome, Piazzale Aldo Moro 5, 00181 Rome, Italy; mariaenrica.dicocco@uniroma1.it (M.E.D.C.); valeria.raguso@gmail.com (V.R.); flavia.milanetti@uniroma1.it (F.M.); maurizio.delfini@uniroma1.it (M.D.); 3 Department of Pharmacy, University of Napoli Federico II, Via D. Montesano 49, 80131 Napoli, Italy; asantini@unina.it

**Keywords:** NMR, metabolomics, *Brassica oleracea* (var. *italica*), organic and conventional pratices, glucosinolates

## Abstract

Brassicaceae family provides several crops which are worldwide known for their interesting phytochemical profiles, especially in terms of content of glucosinolates. These secondary metabolites show several beneficial effects toward consumers’ health, and several studies have been conducted to identify cultivation factors affecting their content in crops. One of the agronomic practices which is attracting growing interest is the organic one, which consists in avoiding the use of mineral fertilizers as well as pesticides. The aim of this study is to define the metabolic profile of *Brassica* *oleracea* (var. *italica*) and to compare the samples grown using organic and conventional fertilization methods. The hydroalcoholic and organic extracts of the samples have been analyzed by NMR spectroscopy. Forty-seven metabolites belonging to the categories of organic acids, amino acids, carbohydrates, fatty acids, sterols, and other molecules have been identified. Thirty-seven metabolites have been quantified. Univariate and multivariate PCA analyses allowed to observe that the organic practice influenced the nitrogen transport, the carbohydrate metabolism, the glucosinolate content and the phenylpropanoid pathway in *B. oleracea* (var. *italica*).

## 1. Introduction

A healthy lifestyle is a combination of behaviors and one of the main aspects is the diet. It is recommended to eat as a minimum five portions of fruits and vegetables daily, thus reducing the risk of chronic disease and improve health outcomes [1]. Vegetables are not only a natural source of amino acids, minerals and vitamins, but they are rich in several secondary plant metabolites that can be subdivided into different groups depending on their chemical structure and functional properties [2]. Some of the secondary plant metabolites with nutraceutical properties [3,4,5] can be found ubiquitously in the entire plant kingdom, and thus, in all types of vegetables. On the other hand, the large and very diverse group of phenolic compounds or carotenoids as well as other secondary plant produced metabolites, are restricted to some botanical orders or families e.g., the glucosinolates (GLs), which are distributed mostly in the order of flowering plants *Brassicales*.

*Brassicaceous* vegetables belong to the order *Brassicale*s and most of them are members of the Brassicaceae family. About the 12% of the world-grown vegetables are *Brassica* vegetables [6], illustrating the great importance of this family. Two very common groups of *Brassicaceou*s vegetables are the *Brassica oleracea* (e.g., broccoli, Brussels sprouts, white and red cabbage, cauliflower, collards, kale, and kohlrabi) and *Brassica rapa* (Chinese cabbage, pak choi, and turnips).

*Brassicaceous* vegetables contain vitamins C, E, and K, as well as folate, minerals, and dietary fiber. *Brassica* generally contains high amounts of vitamin C and can provide up to the 50% of the daily recommended dietary intake of this vitamin [7].

In addition to the phytochemicals, such as carotenoids and phenolic compounds, which occur in considerable amounts in some Brassica species [8,9,10], *Brassicaceous* vegetables are rich also of sulfur-containing compounds e.g., methylcysteinsulfoxide, and glucosinolate [11,12], which are responsible for the pungent and bitter taste or the spicy flavor of *Brassicaceous* vegetables [13,14]. 

One of the most relevant and interest biomolecules in *Brassicales* vegetables are the glucosinolates, due to their health-promoting properties in general, and cancer preventive properties in particular as substantiated by many studies on this topic [15,16,17,18,19].

Glucosinolates are stable secondary metabolite in plants and play a key role in the plant’s defense system. In case of tissue injury (e.g., insect’s damage), they are enzymatically decomposed by the endogenous enzyme myrosinase and, as a result, various degradation products, such as nitriles, epithionitriles, and/or isothiocyanates (ITCs) are released [20]. Isothiocyanates are associated with the pungency of these vegetables and have been shown to confer several beneficial effects [21,22,23], i.e., anti-cancerogenic [2,15,24,25,26,27,28,29,30], anti-inflammatory [31], as well as anti-diabetogenic [32,33] properties and beneficial health effects. 

The composition of secondary metabolites strongly depends on factors such as: (i) pedoclimatic conditions of sampling site; (ii) harvesting time; (iii) plant genotype; (iv) agronomic practices.

Concerning the agronomic practices, the debate about the differences in nutritional properties between organic and conventional food is currently open, as shown by the consistent number of papers and reviews published in the last few years. Comparisons between organic and conventional cultivation methods have shown that organic practices make the plant more susceptible to attacks by pathogens and insects causing an overproduction of secondary metabolites (i.e., phenolic and GLs) in response to the biotic stress respect to conventional. Furthermore, soil fertilization is another factor that can influence the content of plants phytochemicals and the interactive effect depends on crop varieties, plant tissue considered and soil type. As instance, Jones et al. [34] found that nitrogen stress increased glucoraphanin, quercetin and kaempferol content in broccoli florets and decreased glucobrassicin content. The authors hypothesized that the limited nitrogen results in an increased availability of methionine for aliphatic glucosinolate production, while the opposite occurred with tryptophan used for the synthesis of indoyl GLs.

However, the metabolic pathways underlying the biosynthesis of secondary metabolites cannot be analyzed without also studying the whole plant metabolism, which is clearly influenced by soil nitrogen supply.

In recent years, Nuclear Magnetic Resonance (NMR) has emerged as one of the main analytical techniques used in metabolomics. NMR allows to analyze at the same time all the metabolites present in a sample with a single experiment and a minimum of pre-treatment. In fact, the technological advances have allowed to overcome the most important negative problem represented by the low intrinsic sensitivity of this spectroscopic technique. Furthermore, the most advanced two-dimensional techniques allow to identify the compounds present also in extremely complex mixtures, making possible a qualitative and quantitative analysis. For these reasons, NMR spectroscopy is nowadays among the main analytical techniques used in the metabolomics research; it has several advantages including a relatively high degree of reproducibility, easy-to-identify metabolites, high throughput, and non-destructive sample treatment.

On the basis of the metabolic profile obtained from NMR experiments, it has been possible to identify a wide range of metabolites with a single analysis allowing to evaluate various food characteristics, regarding quality, authentication, geographical origin, as well as secondary metabolites with potential nutraceutical properties [35,36,37,38,39].

The aim of this work has been to investigate the effects and interactions of cultivation, organic versus conventional, on the secondary metabolites profile in *B. oleracea* adding two soil fertilizer with different rate of utilization of nitrogen. The two broccoli theses were grown in the same pedoclimatic and soil conditions in order to observe the specific effect of cultivation.

## 2. Materials and Methods

### 2.1. Sampling

For this experiment *B. oleracea* (var. *italica*) cultivar Natalino plants were grown in the Roma (Italy) countryside, in the area of Fiano Romano (Lazio Region, Italy). Natalino variety was selected for its agronomic traits, such as crop robustness, yield stability and an excellent tolerance to freezing stress of both the plant and corymbs. The two cultivation sites were characterized by high similarity in terms of sun exposure and pedoclimatic conditions. The soil is classified as sandy loan with standard mineral dotation of macro and micro elements. The site area is characterized by the Mediterranean climate, with mean annual temperature of 20 °C and annual precipitation of 482 mm concentrated in the Autumn and Spring seasons.

Seedlings were transplanted 6 weeks after germination. Fertilizer were applied at transplanting. Plants were regularly irrigated with drip irrigation system.

Seedlings were transplanted in September and harvested 100 days later in December

Broccoli florets were harvested at the standard commercial ripening stage with characteristic inflorescence consisting of very tight, regular, compact and intense bright green florets with fully developed corymbs. 

Broccoli were grown under organic and conventional agriculture and thirty (30) corymbs from different plants were sampled, fifteen (15) for each of the two types of cultivation. 

No chemicals were used for the control of pests and phytopathological diseases in either conventional or organic cultivations.

Organic and conventional cultivation provided the same amount of nitrogen to the soil and fertilizer applied was urea and bovine manure as reported in Table 1.

After sampling, the samples were put in polyethylene bags at the collection site to avoid water loss, and sent in a refrigerated container to laboratory, where they were stored at −80 °C until further analysis.

### 2.2. Sample Preparation and Metabolites Extraction

Homogeneous portions of the vegetables (0.5 g of fresh weight) were frozen in liquid nitrogen, finely powdered and extracted according to the modified Bligh-Dyer protocol [40]. Each sample aliquot was placed in a mortar, ground in liquid nitrogen and added to a cold mixture composed of with methanol/chloroform/water in a 2:2:1 proportion. The samples were kept at +4 °C for 1 h and then centrifuged for 25 min at 4 °C at 10,000 rpm on an Itettich Zentrifugen centrifuge (Tuttlingen, Germany). This extraction procedure ensures that the metabolic profile does not change and that it is as close as possible to the desired analysis time point. This extraction procedure was employed also because it allows to separate low weight compounds on the basis of their polarity. The upper hydroalcoholic phase and the lower organic one were carefully separated, dried under nitrogen flux and stored at −80 °C until NMR analysis.

### 2.3. NMR Spectroscopy

The hydrophilic phase was resuspended in 0.6 mL of D_2_O containing 3-(trimethylsilyl)-propionic-2,2,3,3-D_4_ acid sodium salt (final concentration of TSP, 2 mM) as an internal chemical shift and concentration standard. The hydrophobic phase was resuspended in 0.6 CDCl_3_ with hexamethyldisiloxane (final concentration of HMDS, 2 mM) as an internal standard. All solvents and standards were purchased from Sigma Aldrich (St. Louis, MO, USA).

All spectra were recorded at 298 K on a Bruker AVANCE III spectrometer operating at the proton frequency of 400.13 MHz and equipped with a multinuclear z-gradient inverse probehead (Bruker BioSpin, GMBH, Rheinstetten, Germany). The ^1^H 1D spectra and 2D ^1^H-^1^H TOCSY, ^1^H-^13^C HSQC and ^1^H-^13^C HMBC were acquired employing previously used parameters [41]. The signals that could be clearly identified and had no overlap with neighboring resonances were integrated for each sample and quantification was performed by comparison of the signal integral with the reference signal, and quantities were expressed in mg/g of fresh weight.

### 2.4. Statistics

Univariate *t*-test analysis was performed with SigmaPlot 14.0 software (Systat Software Inc., San Jose, CA, USA). Multivariate PCA was performed on the data matrix of metabolite concentrations measured by NMR spectroscopy with the Unscrambler ver. 10.5 software (Camo Software AS, Oslo, Norway). Data were mean centered, since the variables with the largest response could dominate the PCA, and then autoscaled to equalize the importance of the variation of each variable.

## 3. Results and Discussion

Comprehensive metabolic profile analysis of *B. oleracea* var *italica* was carried out by ^1^H NMR spectroscopy of hydroalcoholic and chloroform extracts. The extracts of the two cultivation practices showed only quantitative differences and not qualitative ones (Figures S1 and S2 for hydroalcoholic and chloroform extracts, respectively). Resonance assignment was carried through bidimensional TOCSY (Figures S3 and S4), HSQC (Figures S5 and S6) and HMBC (Figures S7 and S8) experiments and confirmed by literature data [41,42]. A total of 47 metabolites were identified; 37 were quantified, and the ^1^H chemical shifts, multiplicity and the ^13^C chemical shifts are reported in Supplementary Table S1. 

The signals that could be clearly identified and had no overlap with neighboring signals were integrated for each sample and quantification was performed by comparison of the signal integral with the reference one, and quantities were expressed in mg/g of fresh weight. The resulting data set was studied by univariate and multivariate statistical analysis tools for the evaluation of statistical differences between the two fertilization methods. The quantitative analysis is reported in Table 2 with the statistical significance assessed by student *t*-test.

Comparing the profile of *B. oleracea* fertilised with only manure with the one fertilised with both urea and manure, it is possible to observe an increase of aspartate, glycine, tyrosine, histidine, malate, sucrose, linolenic fatty acid, total choline, glucoraphanin, and glucobrassicin as well as a decrease of valine, isoleucine, threonine, glutamine, lysine, arginine, asparagine, phenylalanine, acetate, and fructose.

**Figure 1:**
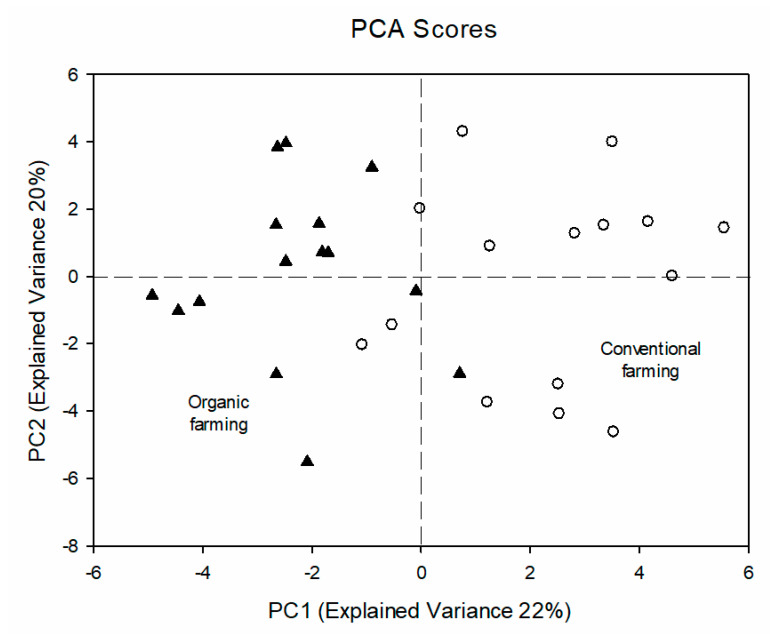
PCA score plot analysis (PC1 vs. PC2) of *Brassica oleracea* (var. *italica*) samples. Conventional samples are indicated by white circle and organic sample are indicated by black triangles (for the abbreviation used see Table S1).

To observe correlations among the quantified molecules, a PCA analysis was carried out on the whole data matrix, providing a model whose first 6 components explained 80% of the overall variance with the first component (PC1) accounting for 22% and the second one (PC2) for 20% of the overall variance as shown in Figure 1. 

**Figure 2:**
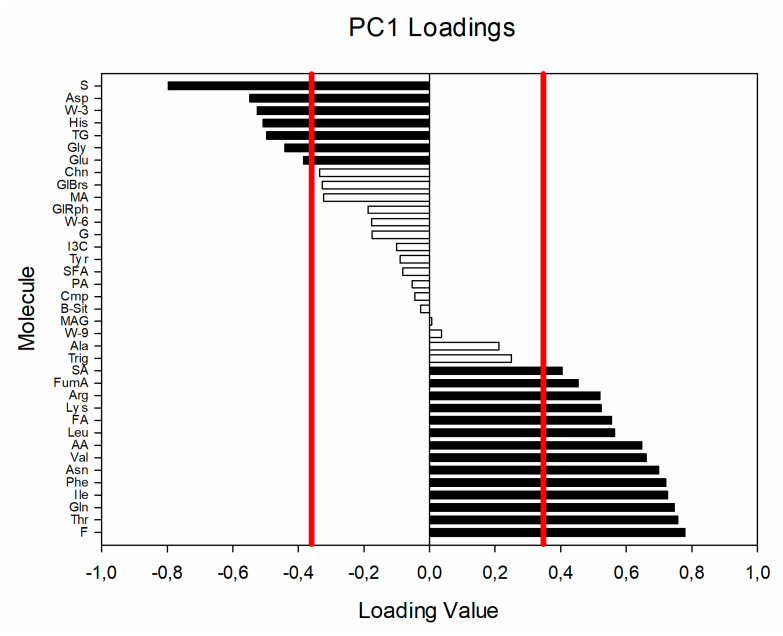
Normalized PC1 loading values. The red lined indicate the significance threshold and in black the variables with *p* < 0.05 are evidenced.

Analyzing the PCA score plot, while there is not a clear grouping of the samples according to the farming, it has been possible to observe that conventional samples were mainly at positive values of PC1, while most of the organic ones were at negative values. A *t*-test performed on the PC1 values of the samples indicated the observed difference between the samples to be significant (*p* < 0.01). The variables important for the discrimination could be determined studying the PC1 loading values (see Figure 2) and normalized loading values greater than 0.349 and lower than −0.349 were considered significant (*p* < 0.05) according to Pearson table for covariance significance.

The molecules negatively correlating with PC1, and thus important for the definition of organic samples were sucrose, aspartate, linolenic fatty acid, histidine, glycerol of triglycerides, glycine, and glutamate, while the ones important for the description of conventional samples were fructose, threonine, glutamine, isoleucine, phenylalanine, asparagine, valine, acetate, leucine, formate, lysine, arginine, fumarate, and succinate.

The results of both univariate and multivariate analysis indicated the same molecules to be discriminant between conventional and organic farming. First of all, it is important to remember/underline that for this experimentation no pesticides and other treatments were employed in any plant, and that the fields were close, meaning that the soil composition and the pedoclimatic conditions were the same. As such, any observed difference could only be caused by the different fertilization method. 

As expected, one of the differences could be ascribed to the nitrogen metabolism. Indeed, the decrease found in organic sample of glutamine, asparagine and arginine, coupled with the increase of glutamate indicated a lowered activity of the nitrogen transport occurring from the roots toward the stem and the flowers of the plant [43,44]. Moreover, while it is not statistically significant to univariate analysis, fumarate covariate with conventional grown broccoli in PCA as shown in Figure 2. This is interesting since one biosynthetic pathway of arginine starts from glutamine and it includes the fumarate production. Since the correlation values of fumarate and arginine are rather close, this could be a further indication of the observed trend that brings to reduction change of nitrogen transport in organic grown broccoli samples. This hypothesis could be explained by the different degree of absorption of nitrogen from urea compared to manure, as reported in literature [45]. 

At the same time, the plant responded to this condition bolstering its defences, such as the content in glucosinolates, glucoraphanin and glucobrassicin, which were higher in organic grown plants. It is interesting to observe that not only the molecules were more abundant, but so were also many of their amino acidic precursors. As reported in the literature, the precursors of the core moiety are glucose, glycine, and methionine [46].

While methionine could not be quantified, both glucose and glycine content increased in organic samples, even if glucose increase was not statistically significant.

The observed variation in the amount of the aromatic amino acid levels, tyrosine and histidine which are precursors of several secondary metabolites belonging to the phenylpropanoid pathway [47,48], their increase in organic broccoli could be interpreted as a bolstering of this pathway. On the other hand, in the same samples phenylalanine, another precursor of phenylpropanoids, was present in a lower amount than in conventional plants as shown in Table 1. This apparent inconsistency could be explained by the fact that phenylalanine is the precursor of both aromatic amino acids as well as other secondary metabolites.

Another indication of the gearing of substrate reprogramming toward defence metabolites could be the increase of linolenic fatty acid. This molecule is the precursor of several volatile metabolites with defensive functions and Brassicaceae family contains several of them [49].

The carbohydrate metabolism was affected also by this adaptation: the increase of sucrose, which is usually coupled with a reduction of fructose levels, has been reported in literature to be in indication of the plant to store energy to be utilized for the production of molecules to be used against pathogens [50]. 

## 4. Conclusions

This study has shown that, in the absence of pathologies, there is a strong response of the metabolism of broccoli grown with organic practice, which stimulates the production of secondary metabolites, as observed by the increase in the concentration of glucosinolates, glucoraphanin, and glucobrassicin, as well as their precursor amino acids.

From the literature it is known that the nitrogen supply greatly influences the glucosinolate content. This is an important aspect for this study, as it allows to hypothesize that the slower release of nitrogen from manure compared to urea affects the plant growth which responds altering the metabolism of nitrogen transport.

## Figures and Tables

**Table 1:** Fertilizer application on conventional and organic fields.

	Fertilizer Application (t/ha)	
	Urea	Bovine Manure
Conventional	0.2	15
Organic	0.0	28

**Table 2:** Composition of *B. Oleracea* var. *italica* determined by NMR spectroscopy.

Molecule		Amount (mg/g)		Change
		Conventional	Organic	
Free Amino acids	Valine	0.248 ± 0.046	0.172 ± 0.016 **	↓
	Isoleucine	0.115 ± 0.023	0.074 ± 0.003 **	↓
	Leucine	0.124 ± 0.028	0.106 ± 0.008	
	Threonine	0.153 ± 0.021	0.122 ± 0.006 **	↓
	Alanine	0.307 ± 0.055	0.307 ± 0.024	
	Glutamate	1.349 ± 0.399	1.794 ± 0.245	
	Glutamine	0.729 ± 0.135	0.521 ± 0.028 **	↓
	Aspartate	0.626 ± 0.197	0.843 ± 0.066 *	↑
	Lysine	0.314 ± 0.066	0.254 ± 0.008 *	↓
	Arginine	2.852 ± 0.508	2.378 ± 0.113 *	↓
	Asparagine	1.151 ± 0.234	0.631 ± 0.056 **	↓
	Glycine	0.528 ± 0.147	0.702 ± 0.128 **	↑
	Tyrosine	0.076 ± 0.010	0.100 ± 0.008 **	↑
	Histidine	0.027 ± 0.007	0.056 ± 0.010 **	↑
	Phenylalanine	0.273 ± 0.067	0.159 ± 0.017 **	↓
Organic acids	Acetate	0.042 ± 0.009	0.026 ± 0.002 **	↓
	Malate	2.552 ± 0.452	3.186 ± 0.175 *	↑
	Pyruvate	0.490 ± 0.209	0.650 ± 0.111	
	Succinate	0.148 ± 0.085	0.101 ± 0.012	
	Fumarate	0.148 ± 0.069	0.089 ± 0.020	
	Formate	0.005 ± 0.001	0.004 ± 0.001	
Carbohydrates	Glucose	4.552 ± 1.077	5.451 ± 0.293	
	Fructose	1.634 ± 0.449	1.041 ± 0.083 **	↓
	Sucrose	2.380 ± 0.752	6.946 ± 0.487 **	↑
Lipids and sterols	β-Sitosterol	0.381 ± 0.067	0.352 ± 0.042	
	Campesterol	0.117 ± 0.043	0.112 ± 0.018	
	Stearic acid	1.849 ± 0.794	1.733 ± 0.271	
	Oleic acid	1.024 ± 0.474	1.163 ± 0.168	
	Linoleic acid	0.673 ± 0.156	0.691 ± 0.071	
	Linolenic acid	1.310 ± 0.252	1.663 ± 0.147 *	↑
	Monoacylglycerol	0.356 ± 0.049	0.367 ± 0.029	
	Triacylglygerol	0.305 ± 0.052	0.362 ± 0.028	
Miscellaneous	Choline	0.337 ± 0.058	0.480 ± 0.053 *	↑
	Glucoraphanin	0.565 ± 0.087	0.708 ± 0.027 **	↑
	Glucobrassicin	0.160 ± 0.052	0.449 ± 0.105 **	↑
	Trigonelline	0.026 ± 0.004	0.027 ± 0.001	
	Indole-3-carbinol	0.017 ± 0.007	0.017 ± 0.003	

Values are mean ± standard deviation (sample number *n* = 15). Level of significance: * *p* < 0.05, ** *p* < 0.01; ↑ and ↓ indicate a significative increase and decrease respectively compared to conventional fertilization.

## Supplementary Materials

The following are available online at https://www.mdpi.com/2304-8158/9/7/945/s1, Figure S1: ^1^H spectrum of *Brassica oleracea* var. *italica* hydroalcoholic extract, Figure S2: ^1^H spectrum of *Brassica oleracea* var. *italica* ^1^H spectrum of *Brassica oleracea* var. *italica* chloroform extract, Figure S3: ^1^H-^1^H TOCSY spectrum of *Brassica oleracea* var. *italica* hydroalcoholic extract, Figure S4: ^1^H-^1^H TOCSY spectrum of *Brassica oleracea* var. *italica* chloroform extract, Figure S5: ^1^H-^13^C HSQC spectrum of *Brassica oleracea* var. *italica* hydroalcoholic extract, Figure S6: ^1^H-^13^C HSQC spectrum of *Brassica oleracea* var. *italica* chloroform extract, Figure S7: ^1^H-^13^C HMBC spectrum of *Brassica oleracea* var. *italica* hydroalcoholic extract, Figure S8: ^1^H-^13^C HMBC spectrum of *Brassica oleracea* var. *italica* chloroform extract, Table S1: Metabolites identified in the ^1^H NMR spectra of the *Brassica Oleracea* var *italica*.
