# Supplementary material for: NMR-Based Metabolomic Comparison of Brassica oleracea (Var. italica): Organic and Conventional Farming
Source: Foods. 2020 Jul 17;9(7):945. doi: 10.3390/foods9070945 (PMC7404451; doi:10.3390/foods9070945)
Supplement: Supplementary file 1 [file foods-09-00945-s001.pdf]

NMR-Based Metabolomic Comparison of *Brassica oleracea* (Var. *italica*): Organic and Conventional Farming

Massimo Lucarini [1,*], Maria Enrica Di Cocco [2], Valeria Raguso [2], Flavia Milanetti [2], Alessandra Durazzo [1], Ginevra Lombardi-Boccia [1], Antonello Santini [3], Maurizio Delfini [2] and Fabio Sciubba [2,*]

[1] CREA—Research Centre for Food and Nutrition, Via Ardeatina 546, 00178 Rome, Italy; alessandra.durazzo@crea.gov.it (A.D.); g.lombardiboccia@crea.gov.it (G.L.B.)

[2] Department of Chemistry, "Sapienza" University of Rome, Piazzale Aldo Moro 5, 00181 Rome, Italy; mariaenrica.dicocco@uniroma1.it (M.E.D.C.); valeria.raguso@gmail.com (V.R.); flavia.milanetti@uniroma1.it (F.M.); maurizio.delfini@uniroma1.it (M.D.)

[3] Department of Pharmacy, University of Napoli Federico II, Via D. Montesano 49, 80131 Napoli, Italy; asantini@unina.it

* Correspondence: massimo.lucarini@crea.gov.it (M.L.); fabio.sciubba@uniroma1.it (F.S.)

Received: 12 June 2020; Accepted: 15 July 2020; Published: xxxxxxx

Abstract: Brassicaceae family provides several crops which are worldwide known for their interesting phytochemical profiles, especially in terms of content of glucosinolates. These secondary metabolites show several beneficial effects toward consumers' health, and several studies have been conducted to identify cultivation factors affecting their content in crops. One of the agronomic practices which is attracting growing interest is the organic one, which consists in avoiding the use of mineral fertilizers as well as pesticides. The aim of this study is to define the metabolic profile of *Brassica oleracea* (var. *italica*) and to compare the samples grown using organic and conventional fertilization methods. The hydroalcoholic and organic extracts of the samples have been analyzed by NMR spectroscopy. Forty-seven metabolites belonging to the categories of organic acids, amino acids, carbohydrates, fatty acids, sterols, and other molecules have been identified. Thirty-seven metabolites have been quantified. Univariate and multivariate PCA analyses allowed to observe that the organic practice influenced the nitrogen transport, the carbohydrate metabolism, the glucosinolate content and the phenylpropanoid pathway in *B. oleracea* (var. *italica*).

Keywords: NMR; metabolomics; *Brassica oleracea* (var. *italica)*; organic and conventional pratices; glucosinolates

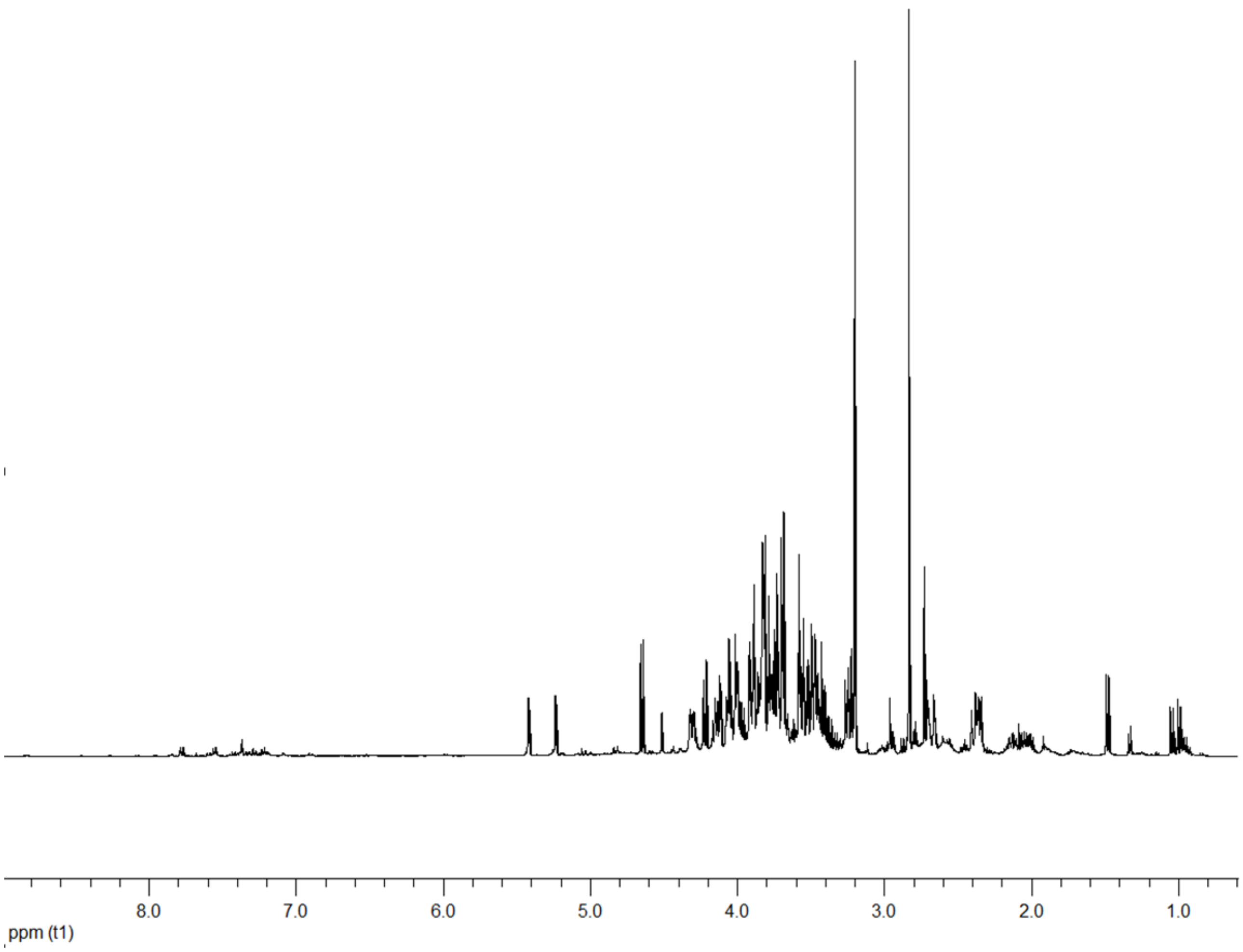

Figure S1: ^{1}H spectrum of *Brassica Oleracea* var *Italica* hydroalcoholic extract

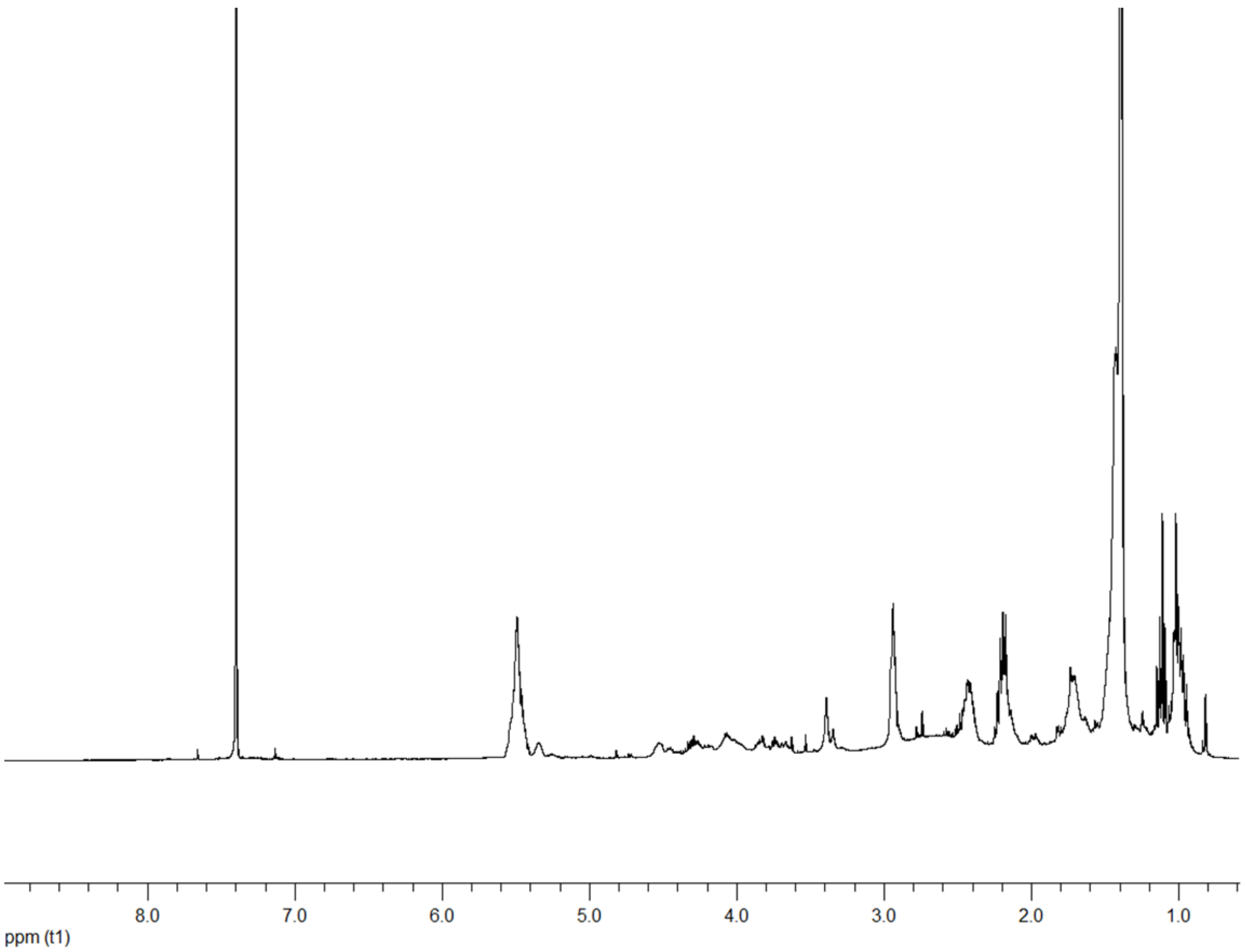

Figure S2: ^{1}H spectrum of *Brassica Oleracea* var *Italica* chloroform extract

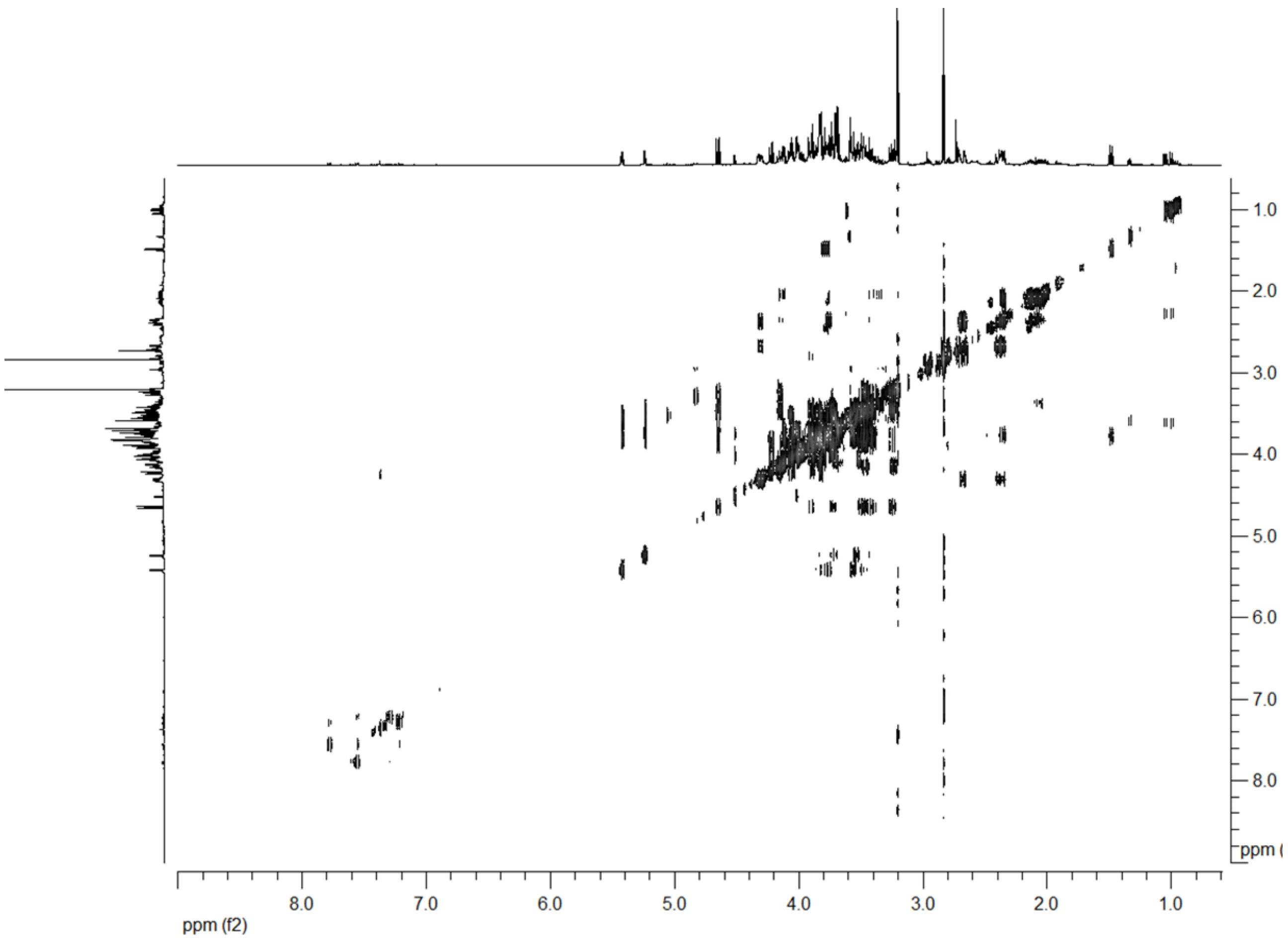

Figure S3: ^{1}H-^{1}H TOCSY spectrum of *Brassica Oleracea* var *Italica* hydroalcoholic extract

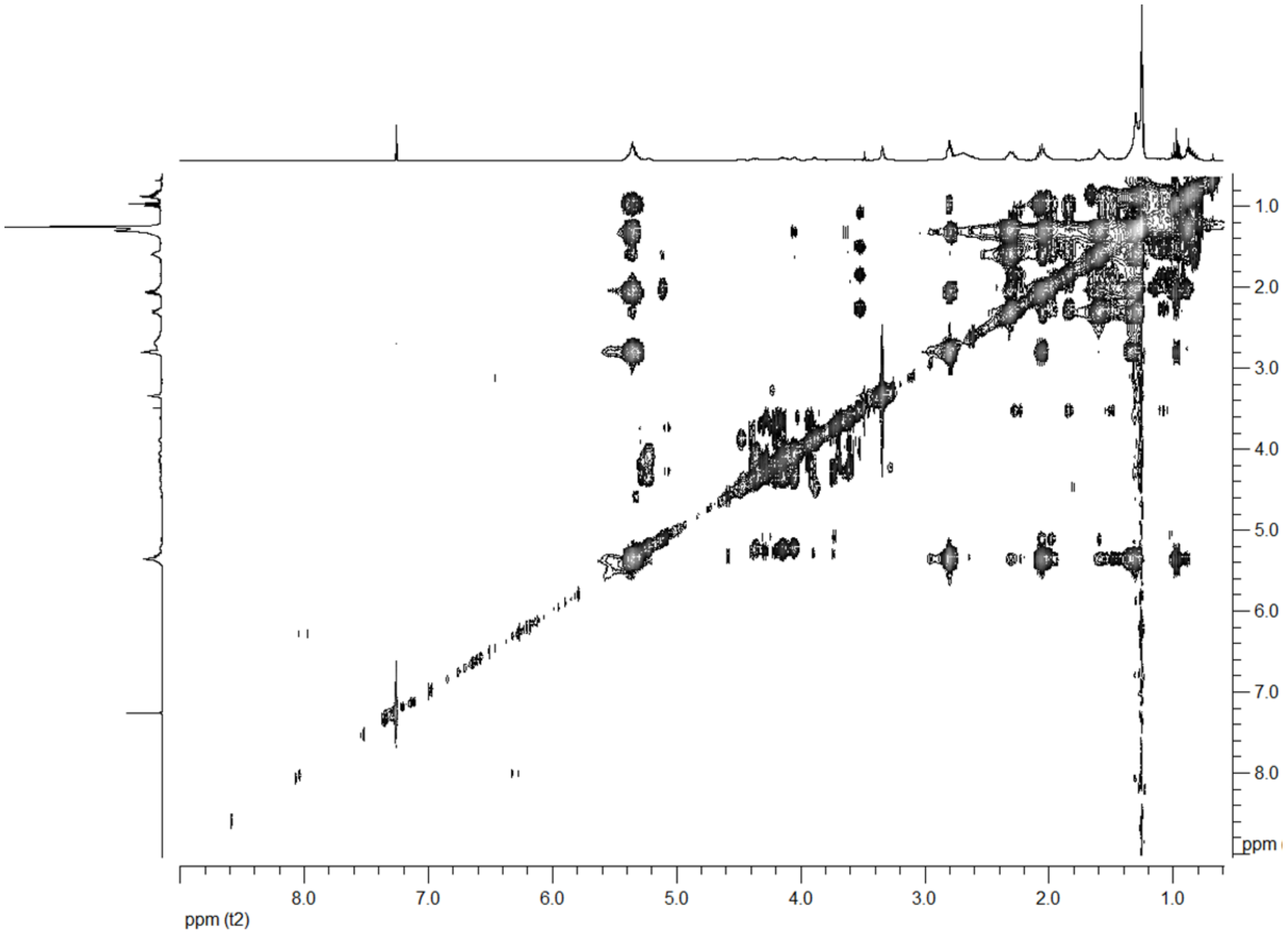

Figure S4: ^{1}H-^{1}H TOCSY spectrum of *Brassica Oleracea* var *Italica* chloroform extract

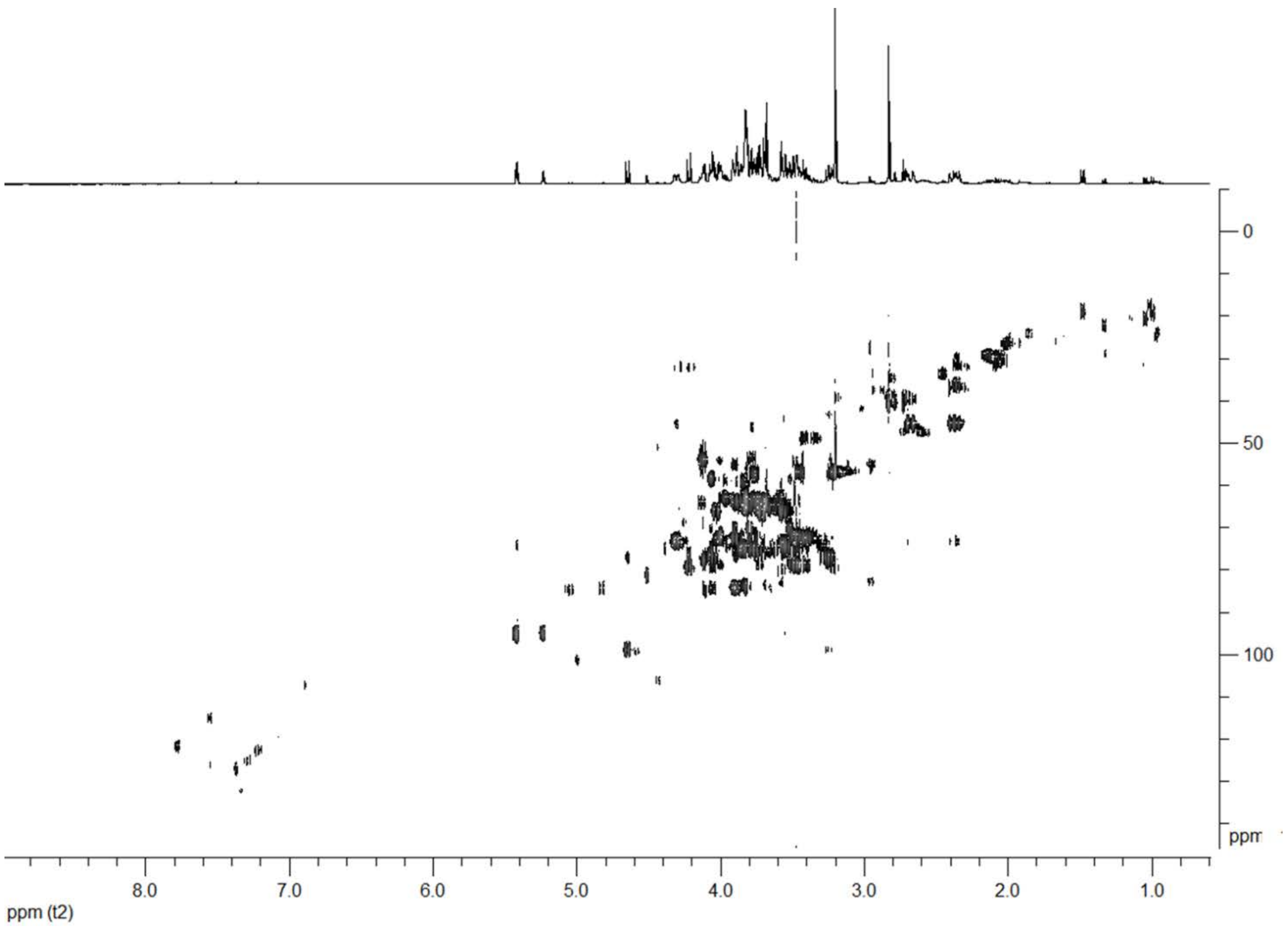

Figure S5: ^{1}H-^{13}C HSQC spectrum of *Brassica Oleracea* var *Italica* hydroalcoholic extract

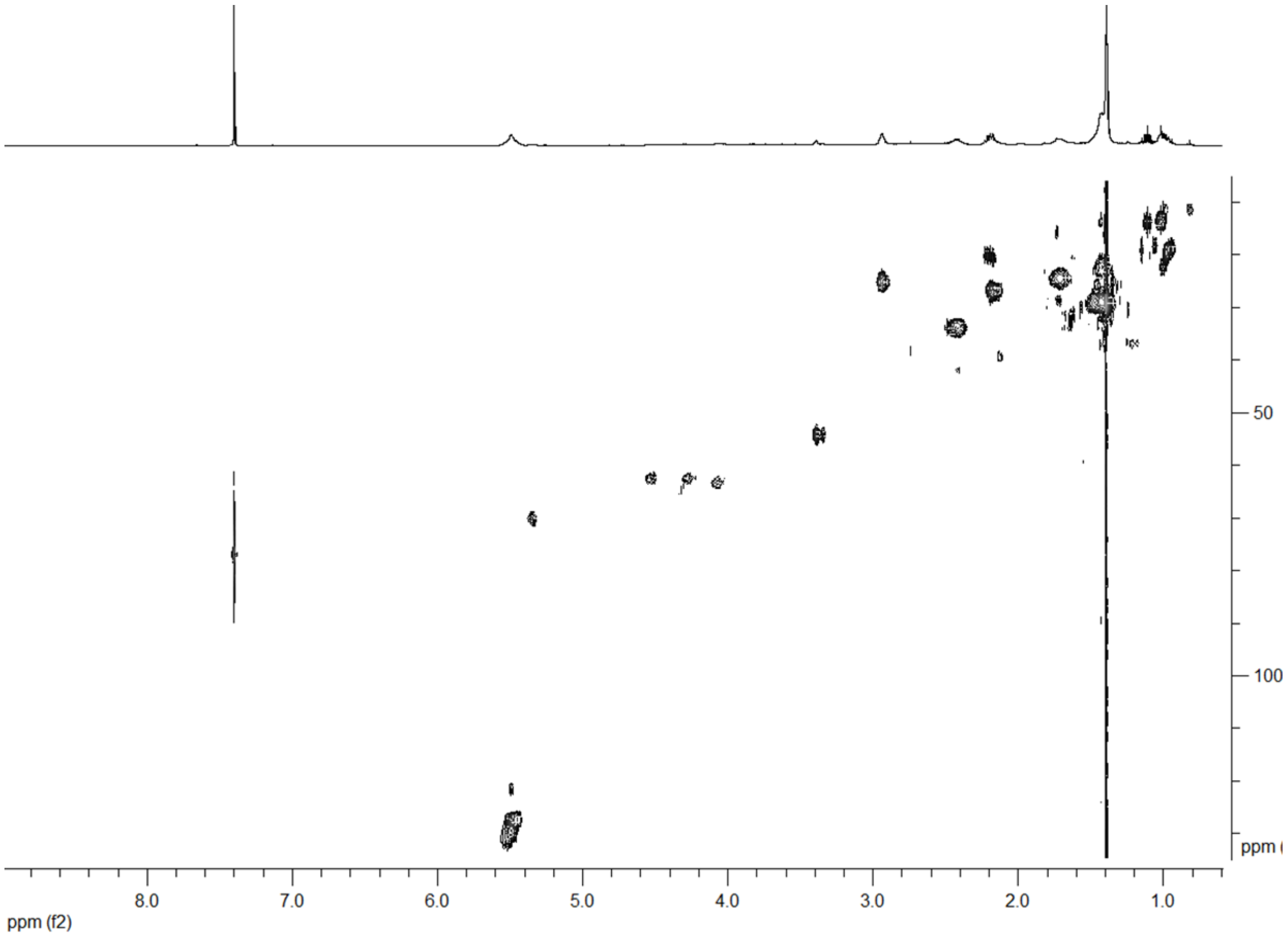

Figure S6: ^{1}H-^{13}C HSQC spectrum of *Brassica Oleracea* var *Italica* chloroform extract

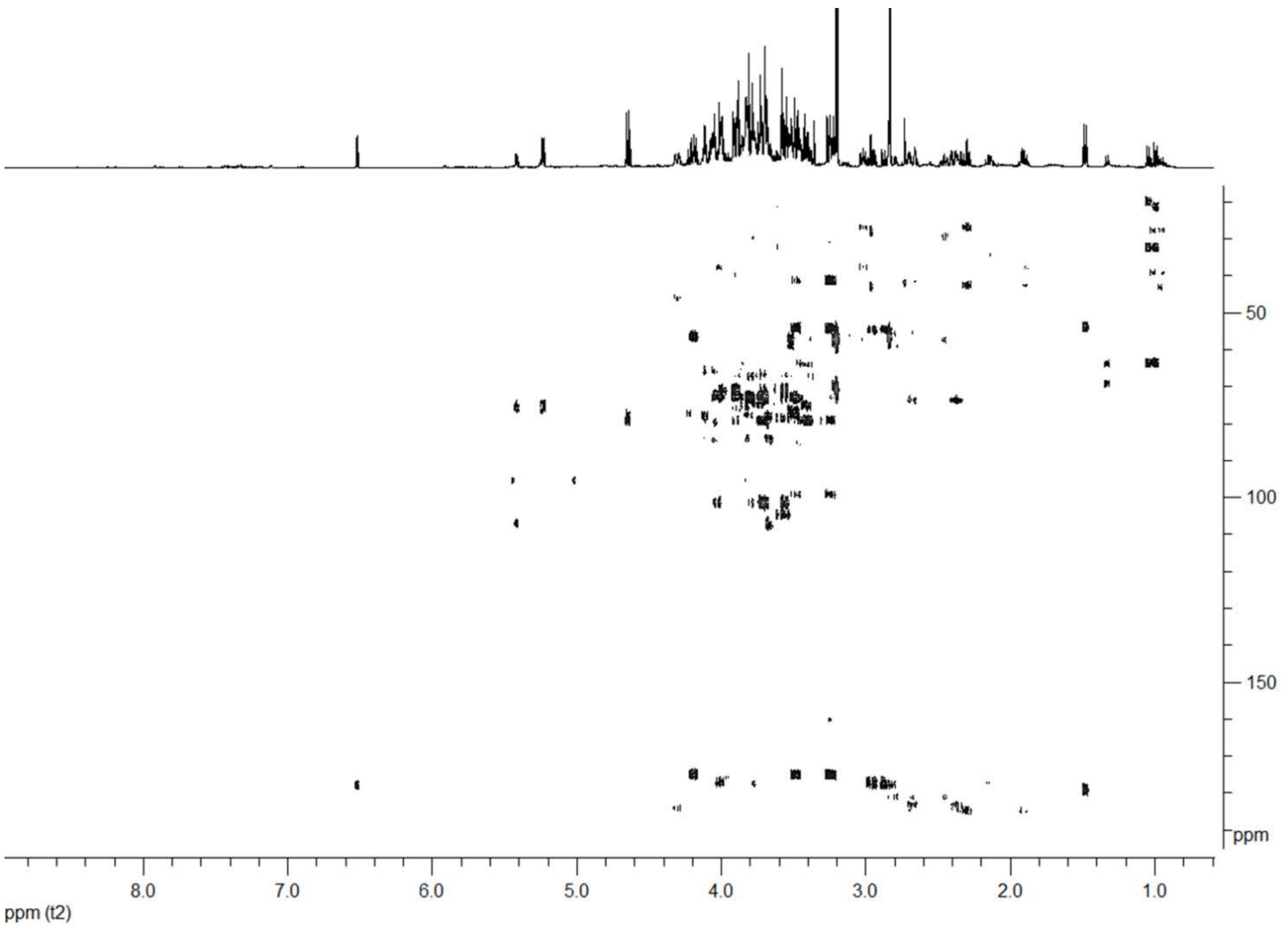

Figure S7: ^{1}H-^{13}C HMBC spectrum of *Brassica Oleracea* var *Italica* hydroalcoholic extract

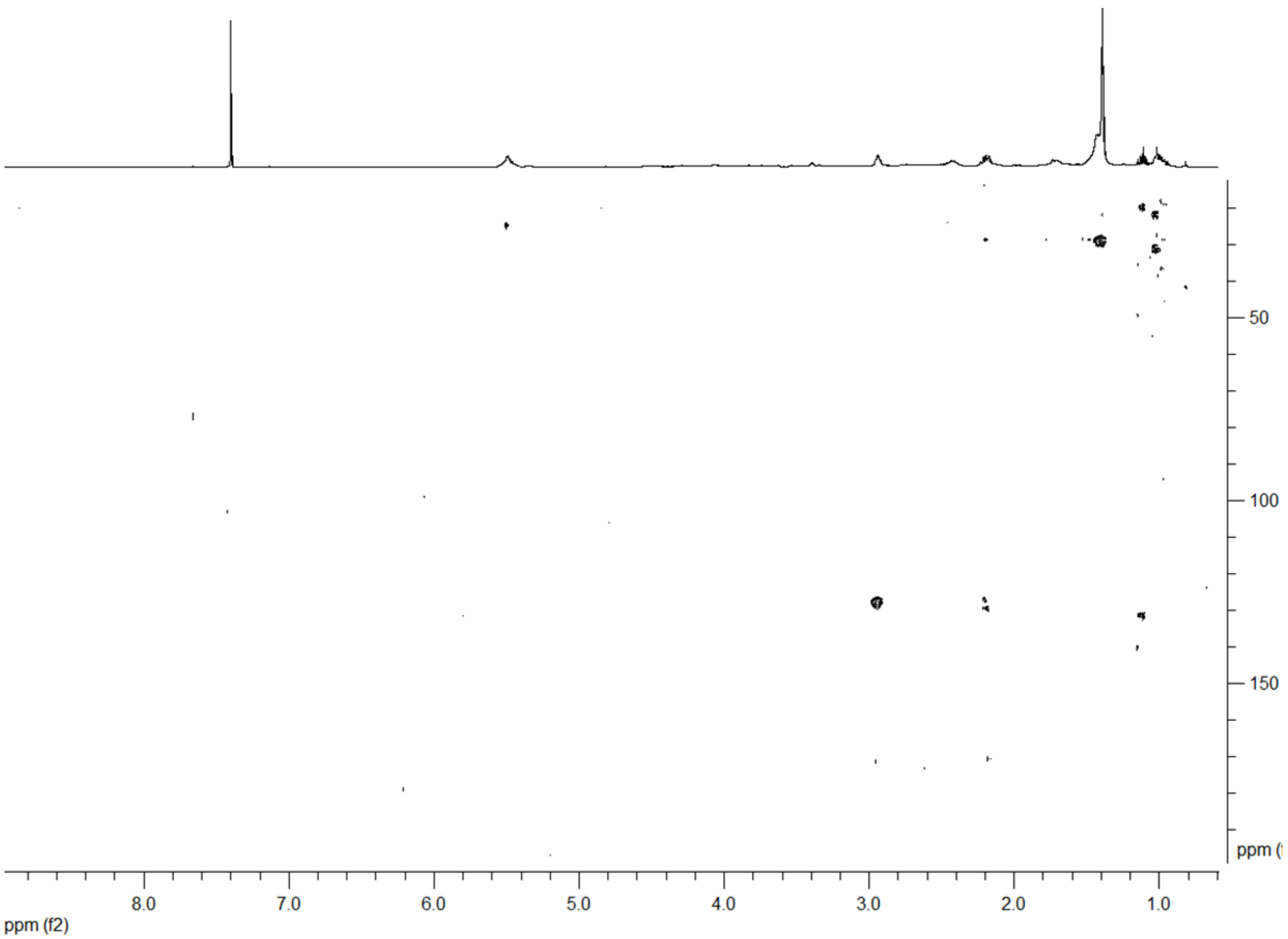

Figure S8: ^{1}H-^{13}C HMBC spectrum of *Brassica Oleracea* var *Italica* chloroform extract

**Compound**	**Assignment**	**δ^1H (ppm)**	**Multiplicity**	**δ^{13}C (ppm)**
**Amino acids**				
**Alanine (Ala)**	**β-CH_3**	**1.49**	d	16.8
	α-CH	3.78	m	51.1
**Arginine (Arg)**	α-CH	3.78	t	54.6
	β-CH_2	1.90	m	28.0
	γ-CH_2	1.71	m	24.4
	**δ- CH_2**	**3.21**	t	41.0
**Asparagine (Asn)**	β-CH	2.87	dd	36.5
	**β'-CH**	**2.96**	dd	36.5
	α-CH	4.01	m	52.6
**Aspartate (Asp)**	β-CH	2.77	dd	37.1
	β'-CH	2.87	dd	37.1
	α-CH	3.92	m	52.7
γ-Aminobutyric acid	β-CH_2	**1.97**	m	24.5
	γ-CH_2	2.42	t	40.1
	α-CH_2	3.03	t	35.2
**Glycine (Gly)**	**α-CH_2**	**3.56**	s	43.6
**Glutamate (Glu)**	β,β'-CH	2.09	m	29.7
	γ-CH_2	2.34	t	36.2
	α-CH	3.73	t	57.6
**Glutamine (Gln)**	**β-CH_2**	**2.11**	m	28.9
	γ-CH_2	2.44	m	33.8
	α-CH	3.78	t	56.9
**Histidine (His)**	β-CH_2	3.16	dd	30.2
	β'-CH_2	3.23	dd	30.2
	α-CH	3.98	dd	57.3
	CH-5	7.09	d	120.0
	CH-2	7.90	d	138.2

**Isoleucine (Ile)**	δ-CH_3	0.93	t	11.8
	**γ’-CH_3**	**1.02**	d	15.4
	γ-CH	1.24	m	25.2
	γ’-CH	1.48	m	25.2
	β-CH	1.95	m	36.6
	α-CH	3.68	m	60.4
**Leucine (Leu)**	**CH_3**	**0.94**	d	21.6
	CH_3'	0.96	d	22.7
	β-CH_2	1.71	m	40.5
	α-CH	3.73	m	54.2
**Lysine (Lys)**	α-CH	3.73	t	54.8
	β-CH_2	1.89	m	30.2
	γ-CH_2	1.43	m	21.9
	δ- CH_2	1.71	m	26.7
	ε- CH_2	3.03	t	39.5
**Phenylalanine (Phe)**	β-CH_2	3.12	m	38.1
	β-CH_2'	3.27	m	38.1
	α-CH	3.98	dd	56.8
	CH-2,6	7.32	d	130.3
	CH-4	7.38	d	128.6
	**CH-3,5**	**7.42**	d	130.6
**Threonine (Thr)**	γ-CH_3	1.33	d	20.0
	α-CH	3.59	m	61.0
	β-CH	4.24	m	66.6
Triptophan	CH-4	7.74	d	120.3
	CH-7	7.54	d	112.8
	CH-6	7.29	m	119.3
	CH-5	7.36	m	122.9
**Tyrosine (Tyr)**	CH-2,6	7.18	d	130.0
	CH-3,5	6.88	d	117.0
**Valine (Val)**	γ-CH_3	0.99	d	17.4
	**γ’-CH_3**	**1.04**	d	18.6

	β-CH<br>α-CH	2.25<br>3.60	m<br>m	29.7<br>61.1
**Organic Acids**				
**Acetic acid (AA)**	**αCH_3**	**1.92**	s	25.9
**Formic acid (FA)**	**$HCOO^-$**	8.45	s	173.77
**Fumaric acid (FumA)**	**α,β-HC=CH**	**6.52**	s	137.9
Isovaleric acid	CH_3, CH_3'<br>CH<br>CH_2	0.91<br>1.94<br>2.04	d<br>m<br>d	24.6<br>28.9<br>50.1
**Malic acid (MA)**	β-CH<br>β’-CH<br>**α-CH**	2.67<br>2.42<br>**4.38**	dd<br>dd<br>dd	43.4<br>43.4<br>71.2
**Pyruvic acid (PA)**	**CH_3**	**2.34**	s	39.2
**Succinic acid (SA)**	**α, β-CH_2**<br>C1,4	**2.39**<br>/	s<br>/	34.8<br>182.9
**Carbohydrates**				
α-Galactose	CH-1<br>CH-2<br>CH-3<br>CH-4<br>CH-5<br>CH6,6'	5.26<br>3.78<br>3.98<br>3.78<br>4.08<br>3.73	d<br>m<br>d<br>m<br>t<br>m	95.1
β-Galactose	CH-1<br>CH-2<br>CH-3<br>CH-4<br>CH-5<br>CH-6,6'	4.59<br>3.50<br>3.62<br>3.97<br>3.72<br>3.82	d<br>dd<br>dd<br>d<br>m<br>m	97.3<br>72.9<br>73.8<br>69.7<br>76.1<br>72.2
**α-Glucose (α-G)**	**CH-1**<br>CH-6<br>CH-3<br>CH-5	**5.25**<br>3.55<br>3.72<br>3.42	**d**<br>m<br>m<br>m	93.1<br>72.5<br>73.8<br>70.7

	CH-2<br>CH_2-6	3.84<br>3.73–3.90	m<br>m	72.5<br>96.9
**β-Glucose (β-G)**	**CH-1**<br>CH-5<br>CH-3<br>CH-4<br>CH-2	**4.69**<br>3.26<br>3.50<br>3.42<br>3.48<br>3.74–3.91	**d**<br>m<br>m<br>m<br>m<br>m	96.9<br>75.2<br>76.8<br>70.7<br>74.6<br>61.8
**Fructose (F)**	CH-1<br>C2<br>**CH-3**<br>CH-4<br>CH-5<br>CH-6	3.57<br>/<br>**4.23**<br>4.10<br>3.82<br>3.79	m<br>/<br>d<br>m<br>m<br>m	63.6<br>102.6<br>76.4<br>75.4<br>81.6<br>63.2
Myo-inositol	CH-1,3<br>CH-2<br>CH-4,6<br>CH-5	3.54<br>4.07<br>3.61<br>3.29	dd<br>t<br>t<br>t	73.3<br>73.1<br>71.9<br>75.1
**Sucrose (S)**	**CH-1**<br>CH-2<br>CH-3<br>CH-4<br>CH-5<br>CH_2-6<br>CH_2-1'<br>C-2<br>CH-3'<br>CH-4'<br>CH-5'<br>CH_2-6'	**5.42**<br>3.59<br>3.79<br>3.48<br>3.85<br>3.82<br>3.69<br>/<br>4.22<br>4.06<br>3.90<br>3.82	**d**<br>dd<br>dd<br>dd<br>m<br>m<br>d<br>/<br>m<br>m<br>m<br>m	93.2<br>72.1<br>73.5<br>70.3<br>73.4<br>61.2<br>62.44<br>104.8<br>77.4<br>75.0<br>82.4<br>63.4
α-Xylose (α-X)	CH-1<br>CH-6<br>CH-3<br>CH-5	5.17<br>3.80<br>3.70<br>3.45	d<br>m<br>m<br>m	93.1<br>72.5<br>73.8<br>70.7

	CH-2	3.35	m	72.5
	CH_2-6	3.73-3.90	m	96.9
β-Xylose (β-X)	CH-1	4.56	d	97.0
	CH-5	3.87	m	74.6
	CH-3	3.69	m	76.8
	CH-4	3.36	m	70.7
	CH-2	3.18	m	75.2
**Miscellaneous**				
**Choline (Chn)**	**-N(CH_3)**	**3.21**	s	56.2
**Glucobrassicin (GlBrs)**	CH-2	7.37	s	126.6
	CH-4	7.55	d	114.9
	CH-5	7.29	t	125.7
	CH-6	7.21	t	122.7
	CH-7	7.78	d	121.8
	CH2-8	4.21	dd	31.9
	CH-1'	4.85	d	82.2
	CH-2'	3.26	m	72.8
	CH-3'	3.41	m	78.2
	CH-4'	3.29	m	69.9
	CH-5'	3.36	m	81.0
	CH-6'	3.62; 3.88	m	61.4
**Glucoraphanin (GlRph)**	CH_2-1	2.75	t	31.6
	CH_2-2	1.92	m	25.5
	CH_2-3	1.87	m	21.5
	CH_2-4	2.82	t	53.0
	CH_3	2.64	s	36.6
	CH-1'	4.85	d	82.2
	CH-2'	3.26	m	72.8
	CH-3'	3.41	m	78.2
	CH-4'	3.29	m	69.9
	CH-5'	3.36	m	81.0
	CH-6'	3.62; 3.88	m	61.4
**Indole-3-carbinol (I3C)**	CH-2	7.21	s	124.7
	CH-4	7.33	d	112.2

	CH-5	7.08	t	121.9
	CH-6	7.01	t	119.7
	CH-7	7.64	d	119.8
	CH-8	4.79	s	57.2
Sinapine	CH_2-10	4.83	m	57.4
	CH_2-11	3.56	m	64.9
	CH-2,6	6.9	s	105.8
	CH-7	7.70	d	146.9
	CH-8	6.47	d	113.3
**Trigonelline (Trg)**	N-CH_3	4.42	s	51.1
	CH-4	8.07	m	130.4
	CH-3,5	8.82	m	148.5
	**CH-1**	**9.11**	s	148.1
**Lipids and Sterols**				
**Stearic Acid (SFA)**	CH_3	0.88	t	14.3
	n-CH_2	1.27	m	29.7
	$C\underline{H}_2$-CH_2-CO_2^-	1.62	m	24.7
	**CH_2-CO_2^-**	**2.31**	**t**	33.9
**Oleic Acid (W-9)**	CH_3	0.88	t	14.1
	n-CH_2	1.27	m	29.3
	**$C\underline{H}_2$-CH=CH**	**2.03**	**m**	27.1
	CH=CH	5.35	m	129.8; 127.4
	$C\underline{H}_2$-CH_2-CO_2^-	1.62	m	24.61
	CH_2-CO_2^-	2.31	t	33.96
**Linoleic Acid (W-6)**	CH_3	0.86	t	14.1
	n-CH_2	1.36	m	29.4
	$C\underline{H}_2$-CH=CH	2.04	m	29.4
	CH=CH	5.37	m	130.3; 128.4
	**=CH-$C\underline{H}_2$-CH=**	**2.76**	**t**	25.7
	$C\underline{H}_2$-CH_2-CO_2^-	2.06	m	24.7

	CH_2-CO_2^-	2.31	t	34.1
**Linolenic Acid (W-3)**	CH_3	0.95	t	14.3
	n-CH_2	1.37	m	29.22
	C<u>H</u>$_2$-CH=CH	2.04	m	27.2
	CH=CH	5.36	m	130.4; 128.3
	**=CH-C<u>H</u>$_2$-CH=**	**2.82**	**t**	26.9
	C<u>H</u>$_2$-CH_2-CO_2^-	2.03	m	24.6
	CH_2-CO_2^-	2.30	t	33.9
**Monoacylglycerol (MAG)**	**CH_2**	**3.65-3.55**	**dd**	65.4
	CH_2	4.05-4.15	dd	70.3
	CH	3.82	m	75.1
**Triacylglycerol (TG)**	CH	5.13-5.21	m	77.4
	**CH_2**	4.15-**4.29**	**dd**	68.2
**Campesterol (Cmp)**	CH_2-1	1.08, 1.85	m	37.2
	CH_2-2	1.51, 1.84	m	31.5
	CHOH-3	3.52	m	71.8
	CH_2-4	2.28	m	42.4
	CH-6	5.34	m	121.8
	CH_2-7	1.52, 1.98	m	32.0
	CH-8	1.46	m	31.8
	CH-14	0.99	m	56.7
	CH_2-15	1.57	m	24.2
	CH_2-16	1.26, 1.85	m	28.4
	**CH_3-18**	**0.70**	**s**	12.2
	CH_3-25	1.01	s	19.1
**β-Sitosterol (β-ST)**	CH_2-1	1.08, 1.85	m	37.2
	CH_2-2	1.51, 1.84	m	31.5
	CHOH-3	3.52	m	71.8
	CH_2-4	2.28	m	42.4

	CH-6	5.34	m	121.8
	CH_2-7	1.52, 1.98	m	32.0
	CH-8	1.46	m	31.8
	CH-14	0.99	m	56.7
	CH_2-15	1.57	m	24.2
	CH_2-16	1.26, 1.85	m	28.4
	**CH_3-18**	**0.68**	**s**	12.2
	CH_3-25	1.01	s	19.1

Table S1: Metabolites identified in the ^{1}H NMR spectra of the *Brassica Oleracea* var *italica*. In bold are evidenced the resonances chosen for metabolite quantification; s: singlet, d: doublet, t: triplet, q: quadruplet, dd: doublet of doublets, m: multiplet.
